# Recurrence of ovarian squamous cell carcinoma with *MET* gene copy number variation: a case report and review of literature

**DOI:** 10.1186/s13048-020-00659-y

**Published:** 2020-05-31

**Authors:** Xuhui Dong, Lei Yuan, Liangqing Yao

**Affiliations:** Department of Obstetrics and Gynecology, Obstetrics and Gynecology Hospital of Fudan University, 128 Shenyang Road, Yangpu District, Shanghai, 200090 People’s Republic of China

**Keywords:** Ovarian squamous cell carcinoma, Optimal cytoreductive surgery, Recurrence, *MET* gene, Copy number variation

## Abstract

**Background:**

Malignant transformation such as ovarian squamous cell carcinoma (SCC) in ovarian mature cystic teratoma (OMCT) is a rare tumor. The gene mutation of ovarian SCC remains unclear. We herein report a recurrent case of ovarian squamous cell carcinoma with *MET* gene copy number variation.

**Case presentation:**

A 60-year-old woman presented with recurrence of ovarian SCC 8 months after primary surgery. Adhesiolysis, right abdominal wall mass excision, prosthetics, enterectomy, enterostomy and partial cystectomy were performed by laparoscope. Pathologic examination demonstrated metastatic squamous cell carcinoma in ileocecus, rectum and abdominal wall muscle. *MET* gene copy number was elevated with copy number of six in this case. Postoperatively, the patient was treated with four cycles of combination chemotherapy with docetaxel and carboplatin. The patient was free of disease at 20 months’ follow-up.

**Conclusions:**

Optimal cytoreductive surgery combined with platinum-based chemotherapy is recommended currently for not only primary tumor but also recurrence. For patients with malignant transformation in OMCT, prompt diagnosis and individualized treatment are crucial for better prognosis. Increased copy number of *MET* may be correlated with her poor PFS and can be a potential therapeutic target for this case.

## Background

Ovarian mature cystic teratoma (OMCT), which is also called dermoid cyst, is a teratoma of a cystic nature that contains kinds of developmentally mature, solid tissues originating from all three germ-cell layers [1]. The incidence of OMCTs is 1.2–14.2 cases per 100,000 people per year and 0.14–2% of them will undergo malignant transformation. More than 80% of malignant transformations are ovarian squamous cell carcinoma (SCC) [2, 3]. OMCT may present at any age, with highest morbidity in reproductive period while SCC in OMTC typically occurs in postmenopausal women. Patients with ovarian SCC often had a dismal prognosis and the stage of the disease was an important factor to the prognosis. The 5-year survival rate for all stages was 48.4%, while for adequately staged patients were 75.7, 33.8, 20.6 and 0% respectively [4]. The appropriate treatment for patients with ovarian SCC remains unsolved. We report a case of a woman with recurrence of ovarian SCC in OMCT and review the literature.

## Case presentation

### Medical history

A 60-year-old woman (gravida1, para1) was referred to our hospital because of recurrence of ovarian SCC in January 2018. She presented with lower abdominal discomfort and transvaginal ultrasonography revealed a 142*115 mm heterogeneous, solid cyst mass in May 2017. Preoperative tumor markers were cancer antigen 125 (CA125): 37 U/ml (< 35) and carcinoembryonic antigen (CEA): 6.18 ng/ml (< 5). Total hysterectomy plus bilateral salpingo-oophorectomy plus omentectomy and iliac lymph node dissection was performed in local hospital due to the malignancy in frozen-section. Pathologic examination indicated right OMCT with malignant transformation into well differentiated SCC; metastases were not found in any other excised specimen. The patient was diagnosed as stage IA according to FIGO classification.

Subsequently, 6 cycles of bleomycin, etoposide and cisplatin was provided (etoposide 0.1 g d1–5, cisplatin 40 mg d2–3, bleomycin 15thousand IU d1–3), and 1 cycle of external beam radiation therapy (EBRT) (DT: 50Gy/25F) as well. During radiotherapy, a mass with diameter of 2 cm on right lower abdominal wall was touched. A biopsy specimen showed well differentiated SCC, ovary origin considered. Then she came to our hospital.

### Auxiliary examination

**Fig. 1:**
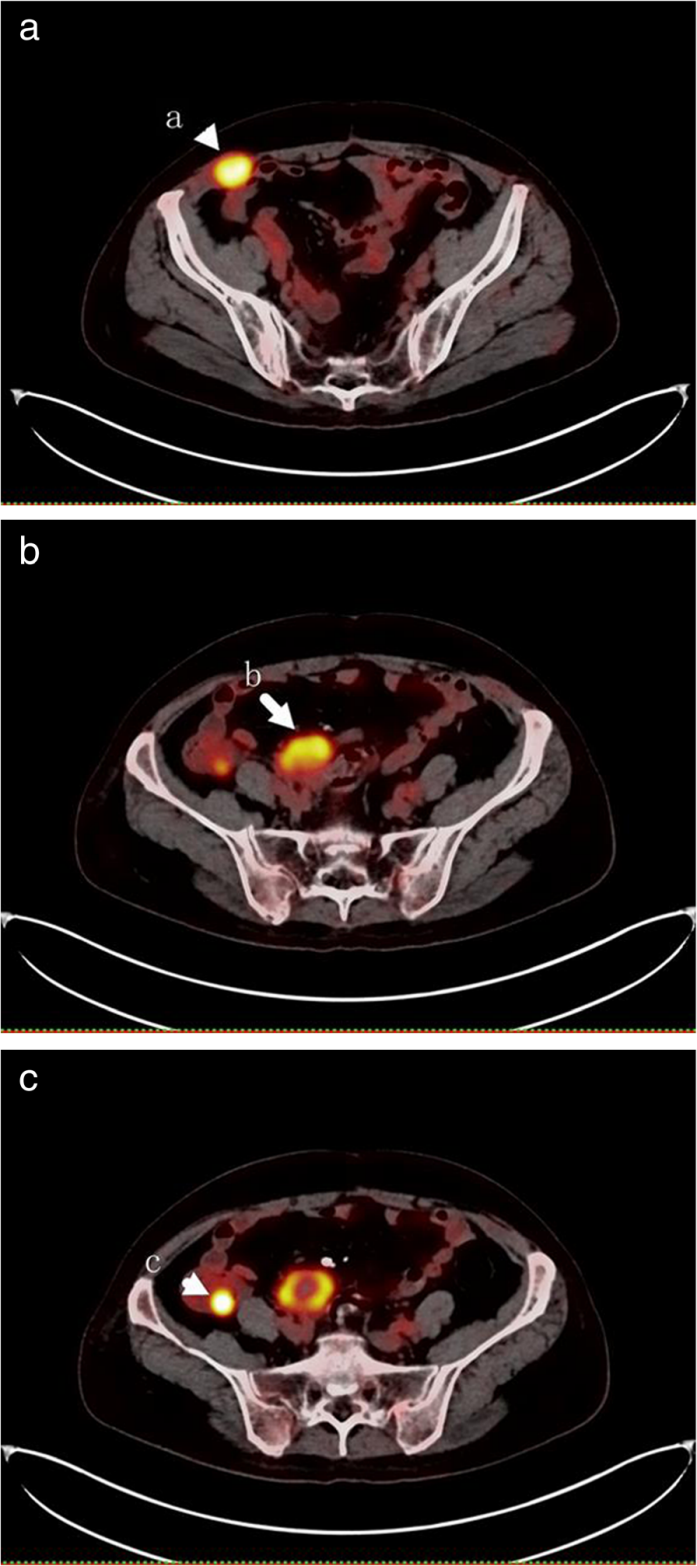
PET-CT fingdings. The elevated uptake of FDG in the right abdominal wall (**a**), multiple soft tissue masses around both iliac vessels (**b**) and ileocecus (**c**)

Positron emission tomography-computed tomography (PET-CT) showed the elevated uptake of ^18^F-Fluorodeoxyglucose (FDG) in right abdominal wall muscle, ileocecus and multiple soft tissue masses around both iliac vessels. (Fig. 1) Elevated tumor marker antigens were human epididymis protein 4 (HE4): 78 pmol/L (< 74.3) and CEA: 6.2 ng/ml (< 5). Other laboratory examinations were within normal values. The patient was human papillomavirus (HPV) negative according to her regular medical examination.

### Secondary cytoreductive surgery and pathological result

**Fig. 2:**
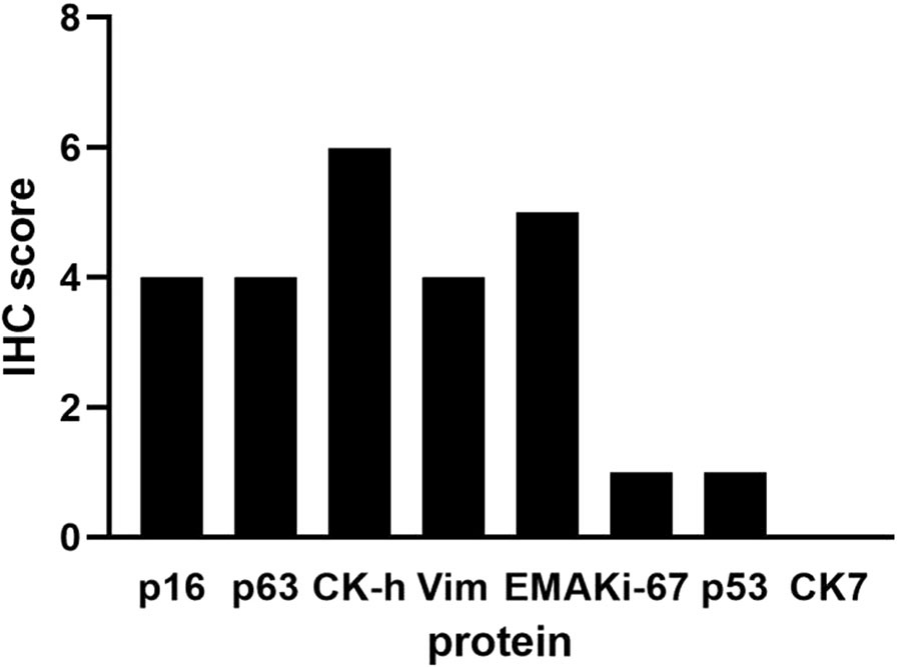
rotein IHC score of secondary cytoreductive surgery. The IHC scores for each protein were calculated and represented as a bar graph. The IHC score was determined by multiplying the score for staining intensity with the score for positive area. The intensity was scored as follows: 0, negative; 1, weak; 2, moderate; 3, strong. The frequency of positive cells was defined as follows: 0, less than 5%; 1, 5 to 25%; 2, 26 to 50%; 3, 51 to 75%; 4, more than 75%

Adhesiolysis, right abdominal wall mass excision, prosthetics, enterectomy, enterostomy and partial cystectomy were performed by laparoscope. 3 tumor masses were detected at the right abdominal wall, ileocecal junction and rectum with a diameter of 3 cm, 7 cm and 4 cm, respectively. Pathologic examination demonstrated metastatic squamous cell carcinoma in ileocecus, rectum and abdominal wall muscle. Immunohistochemically, these cells were diffusely positive for p16, p63, CK-h, Vim and EMA, partly positive for Ki-67 and p53, but negative for CK7. (Fig. 2).

### Whole exome sequencing

The whole exome sequencing identified 396 somatic mutation events in this case. (Table 1) Two hundred and thirty-nine (60.4%) were nonsynonymous single nucleotide variation (SNV) and thirty-four were disruptive insertion-deletion events. There were mutations in 7 known driver genes including *TP53*, *CDKN2A*, *ATR*, *PTCH1*, *MCM-7*, *RAG-1* and *SPTAN-1*. Mutations in *MET*, *MYC* and *JAK2* were all synonymous SNV. Exonic mutation in *PIK3CA*, *KMT2A*, *GNAQ* and *SMARCA4* were not found in this case.

**Table 1:** Classification and number of mutations in exonic region

Type of mutation	number
deletion	28
insertion	6
nonsynonymous SNV	239
stopgain	12
synonymous SNV	111

**Fig. 3:**
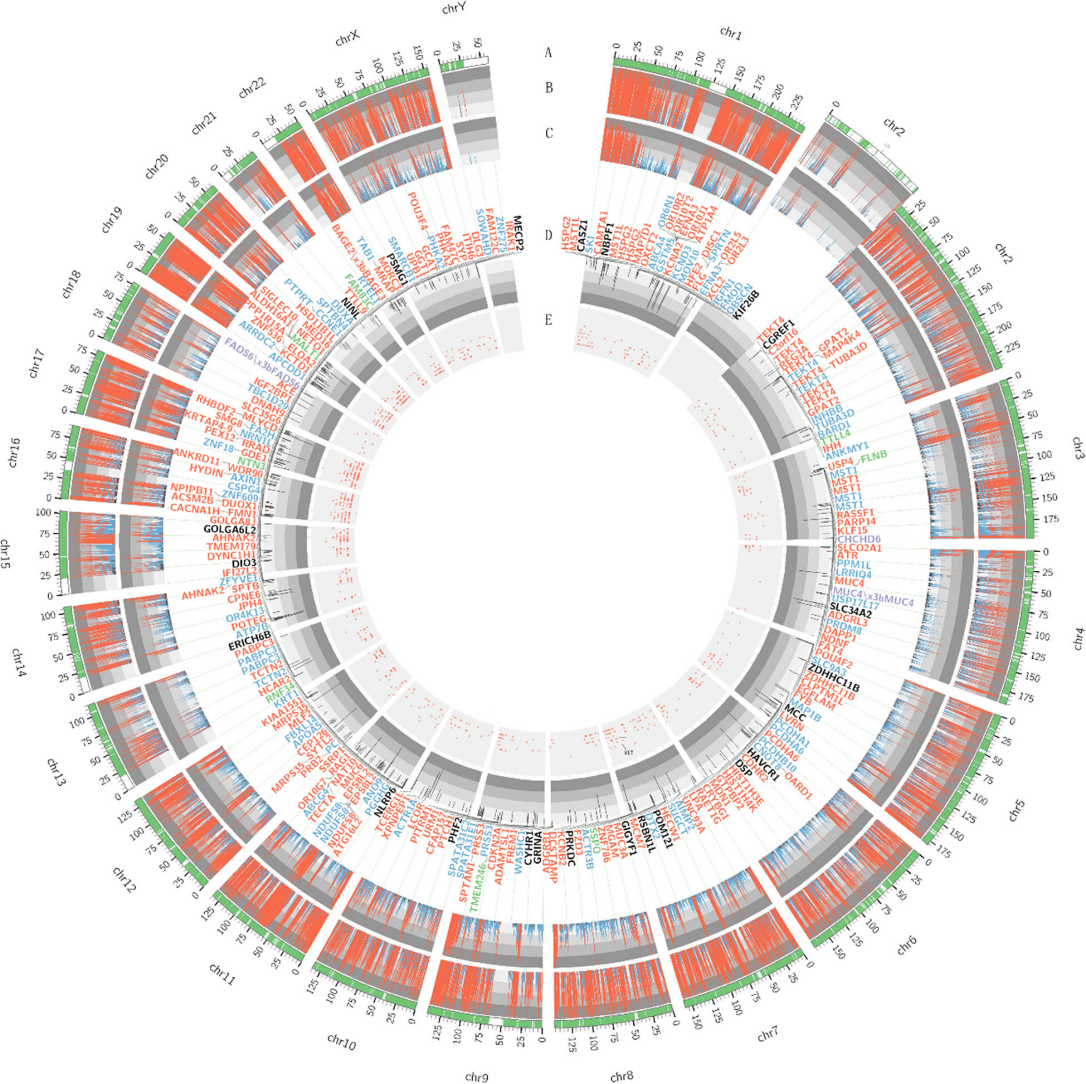
Summary of exome sequencing results displayed as a Circos diagram. Each circle from the outer to inner is: **a** trapping area; **b** sequencing depth of tumor samples: different color represented various depth: red one > = 500, blue one > = 100, others are black; **c** sequencing depth of control group samples; **d** frequency of somatic mutation and gene related to exonic and splicing mutation: splice site mutation, synonymous mutation, nonsynonymous mutation and terminator codon mutation are purple, blue, red and green respectively. **e** somatic cell CNV: various color represents different copy number: red one > 2, black one = 2 and blue = 1

The copy number variation (CNV) was shown in Table 2. Gene amplification in *MYC* and *EGFR* which was reported in previous study were also found in this case. Besides, *MET* gene copy number was elevated with copy number of six which indicated the patient may benefit from c-MET small molecule tyrosine kinase inhibitors such as crizotinib. (Fig. 3).

**Table 2:** Number of copy number variation

Copy number	Number	Interval
1	1	99,464
3	316	385,397,789
4	187	299,076,274
5	153	388,442,175
6	139	628,221,903
7	65	617,883,211
8	26	379,530,248
9	5	53,952,072
11	1	15,567,276
13	1	100,670

### Adjuvant treatment and follow up

After the operation, the patient was offered platinum–based chemotherapy for 4 cycles (carboplatin AUC5, docetaxel 75 mg/m^2^ intravenously). The patient was followed up every 3 months. The patient recovered well, and no side effect was observed during follow-up. PET-CT scan in July 2019 showed no abnormally elevated uptake of FDG. Up till now, there has been no evidence of tumor recurrence.

## Discussion and conclusions

Risk factors of malignant transformation in OMCTs.

Malignant transformation arising from OMCTs is diagnosed with difficulty in the preoperative period. Only 1–2% of them can be diagnosed preoperatively. We can only depend on some risk factors as follow: i) postmenopausal age; ii) diameter more than 10 cm or growing rapidly; iii) elevated serum tumor markers especially SCC antigen and CA125; iv) the presence of a solid component that extends transmurally and invades the adjacent structures in magnetic resonance imaging (MRI) [1, 3, 5–7]. Cases with these factors usually had a worse prognosis [4].

Our patient is a postmenopausal woman with a 142*115 mm mass and mildly elevated the level of tumor markers. Before the primary operation, she didn’t receive further examination such as CT or MRI. Although the frozen-section found a positive result, more auxiliary examination should be done before the primary surgery.

### Treatment of ovarian SCC

Operation played a pivotal role during both diagnosis and treatment. Patients who received optimal cytoreduction followed with adjuvant chemotherapy seem to have a good outcome with longer survival [8, 9]. Optimal cytoreduction can bring a mean survival of 14 months while 7.8 months in suboptimal cases [10]. Kikkawa et al. [6] reported a 79% 5-year-survival rate for patients without residual disease compared with 10.1% for those with residual disease. Minimization of tumor burden may improve the effect of adjuvant treatment. Optimal surgical resection can provide a better curative effect in both initial and recurrent cases.

On account of the activity of cisplatin in ovarian cancer and gynaecological squamous-cell carcinomas, chemotherapy with cisplatin and taxane became a common therapy in SCC, and have been extended progression-free interval in most patients [3, 8, 11, 12]. Patients received adjuvant chemotherapy (mostly platinum-based) appeared to survive longer than those who had surgery only [4, 13]. Several studies suggested that chemotherapy with BEP can be used in ovarian SCC [14]. Nevertheless our patient had a poor effect to the chemotherapy with BEP, which may lead to the recurrence within a short period of time.

### Gene mutation in OMCTs

Several studies showed that malignant transformation of OMCTs may be associated with genetic mutations, high-risk human papillomavirus infection and metaplastic squamous epithelium [15–19]. OMCT-associated SCC had a much higher overall mutational burden and copy number alterations than OMCT. The most frequently altered genes in SCC were *TP53* (20/25 cases, 80%), *PIK3CA* (13/25 cases, 52%) and *CDKN2A* (11/25 cases, 44%) [20].

In the study done by Iwasa et al. [21], overexpression of the p53 protein was observed in 67% SCC cases (14 in 21 cases), while four of them had point mutations in the p53. In humans, p16 is encoded by the *CDKN2A* gene. Expression of p16 protein decreased in 86% patients (18 in 21 cases), and p16 hypermethylation and point mutation was noticed in 6 and 7 respectively. Another study indicated that p53 and p16 expression in SCC were significantly higher than that in the teratomatous skins [22]. P53 and p16-Rb pathways may be related to SCC arising in OMCT. The mutation of *TP53* and *CDKN2A* were also found in our case. Other 5 genes (*ATR*, *PTCH1*, *MCM-7*, *RAG-1* and *SPTAN-1*) were not mentioned in any research about ovarian SCC, although they were demonstrated to be corrected with oncogenesis and chemotherapy resistance in epithelial ovarian cancer or SCC at other sites.

In addition, p16 overexpression are usually caused by the HPV. The role of HPV in the malignant transformation of OMCT is controversial. There were reports that HPV infection might be a factor that lead to the malignant transformation [19, 23]. On the contrary, Cooke et al. found no SCC was positive for HPV in their 25 cases [20]. In our case, we also can find diffusely positive for p16 in immunohistochemical staining while her was HPV negative. More validation studies were needed to explore whether HPV might have acted as the root cause for the malignant transformation in OMCT.

Due to low morbidity, it is still not clear to the oncogenesis of SCC in OMCT. Because of the tumor heterogeneity, the gene mutation which is the base of targeted therapy in carcinoma with same histological type varies from case to case. Gene sequencing may be a facial step to both diagnosis and treatment. Nowadays personalized cancer medicine becomes more and more important during the treatment in malignancies. Except for treatment mentioned above, targeted therapy is also a way for refractory cases. Increased copy number of *MET* in our case may be a potential therapeutic target.

### *MET* gene CNV and the targeted therapy

*MET* gene CNV in ovarian SCC hasn’t been reported in previous studies. The *MET* gene is located on chromosome 7q21-q31 and encodes a receptor tyrosine kinase c-MET for hepatocyte growth factor (HGF) [24, 25]. When bound by HGF, c-Met is activated by autophosphorylation along with a number of phosphorylated proteins, which in turn phosphorylates and activates several downstream pathways [24]. And then a range of biological effects may present in tumor cells including scattering, branching morphogenesis, cell motility, invasion, migration and eventual metastasis [26].

Aberrant c-Met activation is associated with cancer progression such as more sites of metastasis and an adverse outcome in many malignant tumers [27]. Epithelial ovarian carcinomas, which mostly consisted in serous cancer, with c-Met expression might be a potential prognostic marker for patients with advanced stage [28]. Patients with high c-Met levels had a shorter median survival compared with those with low c-Met expression (17 months versus 32 months, *P* = 0.001) [29]. To the contrary, another two studies showed high c-Met cases with clear cell carcinoma and mucinous carcinoma had lower stage as well as significantly longer PFS [30, 31]. The prognosis significance of c-MET may be different between type I and type II ovarian cancer.

CNV of *MET* was a common characteristic in malignant tumor. The high polysomy CNV of the *MET* gene and was found in 18% (16/89) clear-cell adenocarcinomas and 1.88% (3/106) non-clear-cell type ovarian carcinomas [32]. Another study indicated that high polysomy of chromosome 7 was found in 9.5% (10/105) ovarian cancers and most of them were high-grade serous carcinomas [30]. In addition, *MET* amplification was found in several tumors associated with adverse prognostic factors in carcinoma origin from lung, stomach, gallbladder and ovary. Shorter PFS and OS were observed in cases with increased CNV of *MET*. Although malignant transformation in OMCTs with an increased CNV of *MET* had been never reported, her poor PFS met the conclusion mentioned above.

Gene amplification or exon 14 splice site mutations of c-MET in non-small cell lung cancer tumors in which patients may derive benefit from c-MET small molecule tyrosine kinase inhibitors such as crizotinib [33]. Blocking c-Met expression may overcome the resistance of cancer cells to cisplatin [34]. After treatment with crizotinib, cisplatin-induced proliferation inhibition and apoptosis were observed [35]. The long-term efficacy of crizotinib was found in several malignant tumors with *MET* amplification such as gastric and renal carcinoma [36, 37]. A combination of targeted therapy and chemotherapy may be a trend for tumor treatment.

There have been few reports of targeted therapy to patients with ovarian SCC. Verguts et al. [19] reported a case with positive stain for *EGFR*, and she was given gefitinib after bone metastasis. Unfortunately, after 4-month-treatment, disease was progressive. Up to now, there were no signs of recurrence in 15-month follow-up. Due to the poor prognosis of patients with ovarian SCC, we hope to take actions to improve her outcome if her disease progressed and didn’t reflect to standard treatment. At that time, crizotinib may be an alternative therapy after failed multi-line treatment.

In conclusion, ovarian SCC in OMCT is a rare malignancy. Early detection is crucial to patient survival. Whether a primary or recurrent case, complete surgical resection combined with chemotherapy is one of the most important prognosis factors. Individualized treatment is crucial especially for recurrent cases. After optimal debulking surgery and adjuvant therapy, the patient had a good outcome up to now. Increased CNV of *MET* can be a potential therapeutic target. Due to the limited data, further analysis is necessary to elucidate the biology and treatment of SCC transformation in OMCT.

## Data Availability

The data used or analyzed are all included in this published article.

## Abbreviations

OMCT: Ovarian mature cystic teratoma
SCC: Squamous cell carcinoma
CA125: Cancer antigen 125
CEA: Carcinoembryonic antigen
HE4: Human epididymis protein 4
EBRT: External beam radiation therapy
PET-CT: Positron emission tomography-computed tomography
MRI: Magnetic resonance imaging
FDG: Fluorodeoxyglucose
HGF: Hepatocyte growth factor
CNV: Copy number variation
